# Quality Control, Anti-Hyperglycemic, and Anti-Inflammatory Assessment of *Colvillea racemosa* Leaves Using In Vitro, In Vivo Investigations and Its Correlation with the Phytoconstituents Identified via LC-QTOF-MS and MS/MS

**DOI:** 10.3390/plants11060830

**Published:** 2022-03-21

**Authors:** Mohamed S. Abd El Hafeez, Omayma El Gindi, Mona H. Hetta, Hanan F. Aly, Safwat A. Ahmed

**Affiliations:** 1 Department of Pharmacognosy, Faculty of Pharmacy, Egyptian Russian University, Cairo-Suez Road, Badr City 11829, Egypt; omayma-elgindi@eru.edu.eg; 2 Department of Pharmacognosy, Faculty of Pharmacy, Fayoum University, Fayoum 63514, Egypt; mhm07@fayoum.com or; 3 Therapeutic Chemistry Department, National Research Center, Dokki, Giza 12622, Egypt; hanan_abduallah@yahoo.com; 4 Department of Pharmacognosy, Faculty of Pharmacy, Suez Canal University, Ismailia 41522, Egypt

**Keywords:** *Colvillea racemosa*, anti-hyperglycemic activity, α-glucosidase, α-amylase, anti-inflammatory, UPLC-ESI-QTOF-MS, tandem MS/MS

## Abstract

*Colvillea racemosa* is a cultivated ornamental plant that is a monotypic genus of Fabaceae. It is native to Madagascar, with limited studies. For the first time, the leaf quality control parameters, the anti-hyperglycemic and anti-inflammatory in vitro activity of *Colvillea racemosa* ethanol extract (CRE) and its fractions of petroleum ether (CRP), methylene chloride (CRMC), ethyl acetate (CREA), *n*-butanol (CRB), and methanol (CRME) were evaluated. It exhibited significant inhibition against α-amylase, α-glucosidase and membrane stabilization. CRB was the most active fraction, and in vivo studies revealed that oral treatment with CRB of STZ-induced diabetic rats efficiently lowered blood glucose by 67.78%, reduced serum nitric oxide and lipid peroxide levels by 41.23% and 38.45%, respectively, and increased the GSH level by 90.48%. In addition, compared with the diabetic group, there was a 52.2% decrease in serum VCAM, a 55.5% increase in paraoxonase, an improved lipid profile, and improved liver and kidney functions for a treated diabetic group with CRB. Metabolite profiling of CRB was determined by UPLC-ESI-QTOF-MS and tandem MS/MS. Twenty-three chromatographic peaks were identified, which were classified into phenolic compounds and amino acids. The characterized flavonoids were apigenin and luteolin derivatives.

## 1. Introduction

One of the main flowering plant families is Fabaceae. It includes 727 genera and 19,327 species, divided into six recently reclassified subfamilies (Caesalpinioideae, Dialioideae, Detarioideae, Cercidoideae, Duparquetioideae, and Papilionoideae) [1,2,3]. The genus *Colvillea* includes only one species, *Colvillea racemosa* Bojer ex Hook (Colville’s Fame Tree, Whip Tree), native to Madagascar. It is a cultivated ornamental plant, whereas in some tropical regions, it is used as a decorative shade tree [4]. However, to date, biological and chemical investigations of the leaves are rare. Different parts of the *Colvillea racemose* plant showed significant biological activity, as the HPLC analysis of leaves showed flavonoids and phenolic contents which could be responsible for its antioxidant, cytotoxicity, and anti-microbial activity [5], while the isolated compounds from the stem, lupeol, kaempferol, genkwanin, vitexin, aromadendrin, naringenin, isoliquiritigenin, R-liquiritigenin, isovitexin, vicenin 2 and α,β-dihydroxydihydrochalcones *(colveol A, colveol B)*, had high activity as monoamine oxidase inhibitors [6]. On the other hand, 6-methoxy 7-hydroxy bis- coumarin was identified and isolated from *Colvillea racemosa* seeds [7].

Diabetes mellitus is the third most common reason for mortality, behind cancer and cardiovascular disease, with global prevalence [8]. It is harmful to human health and causes significant adverse effects in all parts of the body. Inflammation and oxidative stress, besides insulin resistance, all contribute to a similar pathophysiological signaling cascade for type II diabetes mellitus [9]. According to previous studies, oxidative stress and elevated inflammatory progression frequently combine and are important causes and risk factors for type II diabetes mellitus [9,10,11].

Oxidative stress is characterized by an imbalance in the body between free radicals and antioxidants, leading to the destruction of cells and tissues. Oxidative stress can promote inflammation, which generates excess free radicals, promoting further oxidative stress and creating a destructive cycle [12]. Chronic oxidative stress-induced inflammation can lead to a variety of human diseases, including diabetes and cardiovascular disease, as well as cancer and neurological diseases. Some anti-diabetic drugs can have serious problems. Thus, scientists are currently trying to find naturally antidiabetic compounds with low toxic effects [13].

This study was conducted to evaluate the nutritional value, mineral composition, and anti-hyperglycemic and anti-inflammatory activity of CRE and its fractions (CRP, CRMC, CREA, CRB, and CRME) using several in vitro assays, including α-glucosidase, α-amylase, and membrane stability inhibition activity followed by an in vivo investigation of the most active fraction and the identification of its phytoconstituents by using UPLC-ESI-QTOF-MS.

## 2. Results and Discussion

### 2.1. Quality Control Parameters for Colvillea racemosa Leaves

#### 2.1.1. Proximate Composition

Proximate analysis is an important criterion for classifying a food’s nutritional value [14]. According to the results shown in Table 1, the dominant components are carbohydrate (49.03%), protein (26.23%), and ash (13%), showing a higher mineral concentration in the leaf and lipid content (11.74%); nevertheless, the moisture content was significantly lower (8.2%).

The results reveal that *Colvillea racemosa* leaves are a valuable source of mineral elements due to large quantities of ash. The moisture percentage is less than 15%, implying that leaves are well suited for formulation, since a low moisture content inhibits microbial growth [15]. Leaves are an abundant source of proteins, carbohydrates, and fats, which are the main components of the diet [16]. Because the nutritious value of leaves is considerably higher, they could also give a high energy content. Generally, proximate analysis shows that *Colvillea racemosa* leaves have the possibility to be a dietary supplement.

#### 2.1.2. Minerals

From the results of the analysis presented in Table 2, the levels of calcium (Ca), potassium (K), and magnesium (Mg) content were the highest, at about 2426.67, 2001.9, and 344.01 mg/100 g, respectively. However, other elements were found in small amounts, while selenium (Se), lead (Pb), and cadmium (Cd) were not detected, but when our results were compared with RDI, we found that Ca and manganese (Mn) were above RDI.

The findings reveal that *Colvillea racemosa* leaves have a high concentration of components (Mg, Mn, Ca, and K) that may perform an essential part in the management and control of diabetes mellitus. Because metabolic syndromes, besides type II diabetes, are linked disorders defined by chronic, low-grade inflammation, caused in part by Mg deficiency [19], Mg supplementation has been revealed to increase insulin secretion [20], enhance insulin sensitivity and be a glycemic regulator in Mg-deficient persons [21].

Mn is a cofactor of the antioxidant enzyme catalase and its absence can also lead to reduced activity, leading to free radical accumulation [22]. Mn is a vital element which plays a role in glucose regulation and lipid metabolism in humans. Furthermore, it is required for Mn- superoxide dismutase, which is responsible for ROS in mitochondrial oxidative stress [23].

Epidemiological investigations have also revealed that dietary Ca deficiency is combined with a higher incidence of type II diabetes mellitus, while six weeks of Ca–vitamin D co-supplementation may decrease biomarkers of oxidative stress and inflammation [24]. In addition, K intake affected the incidence of diabetes mellitus [25].

### 2.2. Biological Assessment of the Anti-Hyperglycemic and Anti-Inflammatory Activity of Colvillea racemosa Leaves

#### 2.2.1. In Vitro Anti-Hyperglycemic Assessment via α-Glucosidase and α-Amylase Inhibition Activity Assays

**Figure 1:**
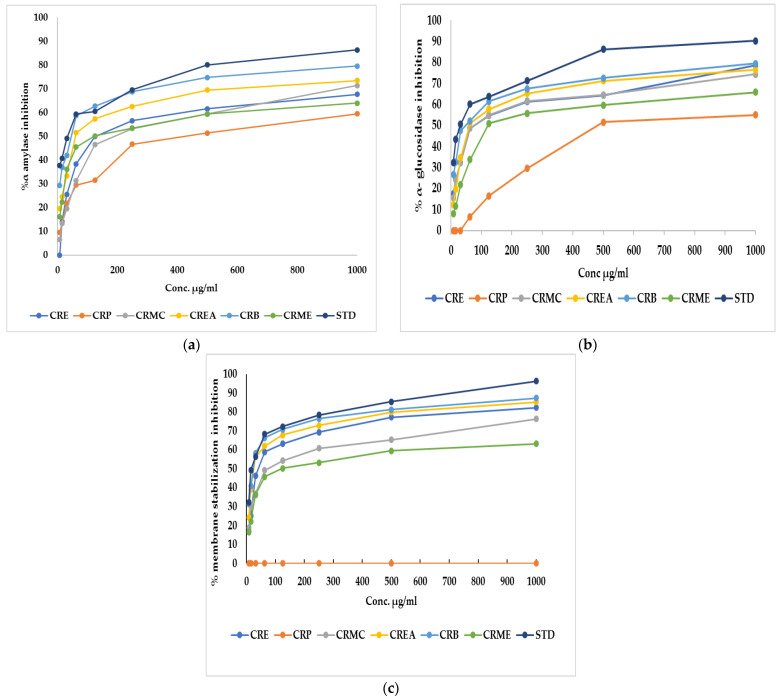
(**a**) α-Amylase inhibition percentage, (**b**) α-glucosidase inhibition percentage, and (**c**) membrane stabilization inhibition percentage of *Colvillea racemosa* ethanol extract (CRE) and its fractions (petroleum ether (CRP), methylene chloride (CRMC), ethyl acetate (CREA), n-butanol (CRB), and methanol (CRME)). STD (acarbose (**a**,**b**)and indomethacin (**c**)).

α-Amylase is the main enzyme which breaks down the polymeric substrate into smaller oligomers by promoting the α-1,4-glucan linkage hydrolysis in starch. It helps catalyze the early stages through the method of converting starch to maltose, then to glucose [26]. Likewise, α-glucosidase is an important enzyme found at the brush border of the human intestinal mucosal, which promotes the hydrolysis of the linear α-1,4-glycosidic bond with the simultaneous generation of glucose [27]. To manage postprandial hyperglycemia, the technique of limiting carbohydrate digestibility by monitoring the action of two hydrolyzing enzymes (α-amylase and α-glucosidase) is considered a promising treatment for diabetes mellitus [26]. Regarding α-amylase, CRE, CRP, CRMC, CREA, CRB, and CRME showed inhibitory potential versus α-amylase, with an IC_50_ of 124.2 ± 1.61, 426.04 ± 0.82, 192.13 ± 1.13, 60.18 ± 0.47,46.6 ± 0.35 and 120.7 ± 1.7 µg/mL, respectively, whereas acarbose, a standard anti-hyperglycemic drug, had an IC_50_ of 34.71 ± 0.24 µg/mL. Concerning α-glucosidase, CRE, CRP, CRMC, CREA, CRB, and CRME displayed inhibitory potential with IC_50_ estimated by 70.8 ± 0.75, 480.3 ± 0.94, 74.9 ± 0.45, 60.2 ± 0.37, 47.7 ± 0.12, and 121.9 ± 0.57 µg/mL, where acarbose displayed IC_50_ of 30.57 ± 0.17 µg/mL The highest inhibition against amylase and glucosidase was observed in CRB. Figure 1a,b show the percent inhibition of different fraction concentrations against α-amylase and α-glucosidase.

#### 2.2.2. In Vitro Anti-Inflammatory Activity Evaluation by Membrane Stabilization % Assay

CRE fractions also exhibited a membrane stabilization effect via preventing hypotonicity-initiated erythrocyte membrane lysis. The erythrocyte membrane is like the lysosomal membrane, so the stabilization of the erythrocyte membrane is related to the stabilization of the lysosomal membrane [28]. The stabilization of the lysosomal membrane is important to prevent the release of chemical mediators and lysosomal components of activated neutrophils, which restrict inflammatory progression [29]. Hypotonicity-induced hemolysis can result from cell shrinkage because of intracellular electrolyte and fluid component osmotic loss. Our extract may inhibit the mechanisms that induce or increase the efflux of certain intracellular components [28]. The effect of CRE and fractions of *Colvillea racemosa* leaves on RBC membrane stability was investigated. It was found that CRE and fractions except for CRP at different concentrations can stabilize the RBC membrane in hypotonic solution and inhibit hemolysis. CRB had similar activity to indomethacin, a reference standard, with an IC_50_ estimated at 23.8 ± 0.14 and 17.02 ± 0.23, respectively. CRE, CRMC, CREA, and CRME, on the other hand, displayed membrane-stabilizing action, with IC_50_ values of 39.9 ± 0.23, 72.14 ± 0.34, 25.5 ± 0.23, and 120.7 ± 0.45, respectively. Figure 1c shows the percent inhibition of different fraction concentrations versus membrane stability.

In the current research, only CRB was evaluated for further studies (in vivo and LC-MS/MS analysis) because the CRB fraction showed the highest inhibitory effect against α-glucosidase, α-amylase, and hypotonicity-induced membrane lysis, which could be attributed to high phenolic and flavonoid contents extracted in *n*-butanol solvent saturated with water [30,31].

#### 2.2.3. In Vivo Anti-Hyperglycemic, Anti-Inflammatory, and Antioxidant Activity Assessment

##### In Vivo Pancreatic Activity Evaluation Using Streptozotocin (STZ)-Induced Hyperglycemia in Rats

Determination of Fasting Blood Glucose (FBG) and α-Amylase Activity

STZ was used to induce hyperglycemia in rats to investigate anti-hyperglycemic activity in vivo. It acts as a diabetogenic agent by increasing the production of ROS in the pancreatic cell [32]. Pancreatic α-amylase is one of the most common amylase enzymes. Suppressing α-amylase enzyme activity helps to reduce hyperglycemia [33].

**Figure 2:**
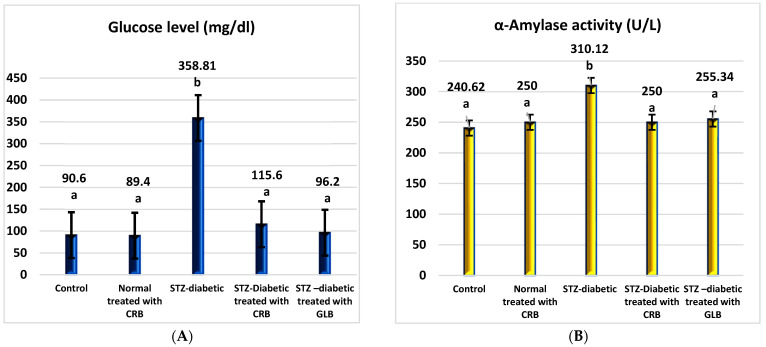
Effect of the n-butanol fraction of *Colvillea racemosa* total extract (CRB) oral administration on the pancreatic activity in diabetic STZ-treated rats: glucose (**A**) and α-amylase activity (**B**). Results are shown as means ± S.D. (measured in triplicate; n = 3). Means that have different letters are significantly different (*p* < 0.05).

As shown in Figure 2, the diabetic group had a significant rise in fasting blood glucose levels of 296.04% compared with the untreated normal group (*p* < 0.05), with a concurrent increase in α-amylase activity of 28.88% compared with the normal group.

The oral administration of glibenclamide (GLB) ameliorates hyperglycemia, manifested as an effective lowering of FBG of approximately 73.19% (*p* < 0.05), with a significant decrease in serum α-amylase activity of 17.66% concerning STZ-induced diabetic rats. Similarly, the treatment of diabetic rats orally with CRB successfully reduced serum blood glucose by 67.78%. This concurrently accompanied a substantial decrease in serum α-amylase activity by 19.39%, greater than that of the GLB-treated group, which reflects the effectiveness of CRB in improving pancreatic activity and hyperglycemia.

##### In Vivo Lipid Profile Assessment Using STZ-Induced Diabetic Rats

Short-term initiation of diabetes using STZ in rats significantly changed the lipid profile when compared with healthy rats. We examined total lipid, triglyceride, cholesterol, and HDL-cholesterol levels to assess the effect of CRB in the lipid profile.

The diabetic group displayed a rise in the levels of total lipid, triglyceride, and cholesterol by 58.54%, 53.7%, and 56.42%, respectively, compared with the control group (*p* < 0.05), with a related reduction in HDL-cholesterol by 39.92%, as shown in Table 3.

In comparison to diabetic rats, the oral administration of GLB effectively improves the lipid profile, as evidenced by a significant reduction in total lipid, triglyceride, and cholesterol levels of 26.31%, 25.11%, and 35.12%, respectively (*p* < 0.05), in addition to a significant increase in the serum HDL-cholesterol level of 76.41%. Meanwhile, the treatment of diabetic rats orally with CRB efficiently decreased serum total lipid, triglyceride, and cholesterol levels by 32.86%, 20.89%, and 29.71%, respectively, compared with the STZ-induced diabetic group, which significantly increased serum HDL-cholesterol by 72.76%.

##### In Vivo Liver Function Assessment Using STZ-Induced Diabetic Rats

Diabetes has a significant impact on various endogenous organs, with the liver being one of the most critical [34]. The most prominent causes of liver damage in diabetic individuals are hyperglycemia-initiated oxidative stress and consequent disturbances in carbohydrate, protein, and lipid metabolisms [35].

The diabetic group showed a rise in ALT, AST, ALP, and bilirubin levels by 110.63%, 59.88%, 72.57%, and 100%, respectively, compared with the control group (*p* < 0.05), with a reduction in total protein level by 34.35% compared with the control group, as shown in Table 4.

In comparison to diabetic rats, the administration of GLB orally successfully ameliorated liver function, manifested as an effective reduction in ALT, AST, ALP, and bilirubin by 47.52%, 39.27%, 41.24%, and 45.83%, respectively (*p* < 0.05), conveyed by a significant increase in serum total protein level of 40.59%. Otherwise, the treatment of diabetic rats orally with CRB effectively decreased serum ALT, AST, ALP, and bilirubin by 43.41%, 31.12%, 39.13, and 41.67%, respectively, compared with the diabetic group, which showed a significantly increased serum total protein level by 43.05%.

##### In Vivo Kidney Function Assessment Using STZ-Induced Diabetic Rats

The diabetic group’s creatinine and urea levels increased by 88.46% and 85.29%, respectively (*p* < 0.05), as shown in Table 5, compared with a control group. In comparison to diabetic rats, the oral administration of GLB effectively improves kidney function, as evidenced by a significant reduction in creatinine and urea of 48.97% and 44.63%, respectively (*p* < 0.05), while treatment of STZ-induced diabetic rats orally with CRB decreased serum creatinine and urea levels by 40.82% and 40.22%, respectively.

##### In Vivo Oxidative Stress Markers Assessment Using STZ-Induced Diabetic Rats

**Figure 3:**
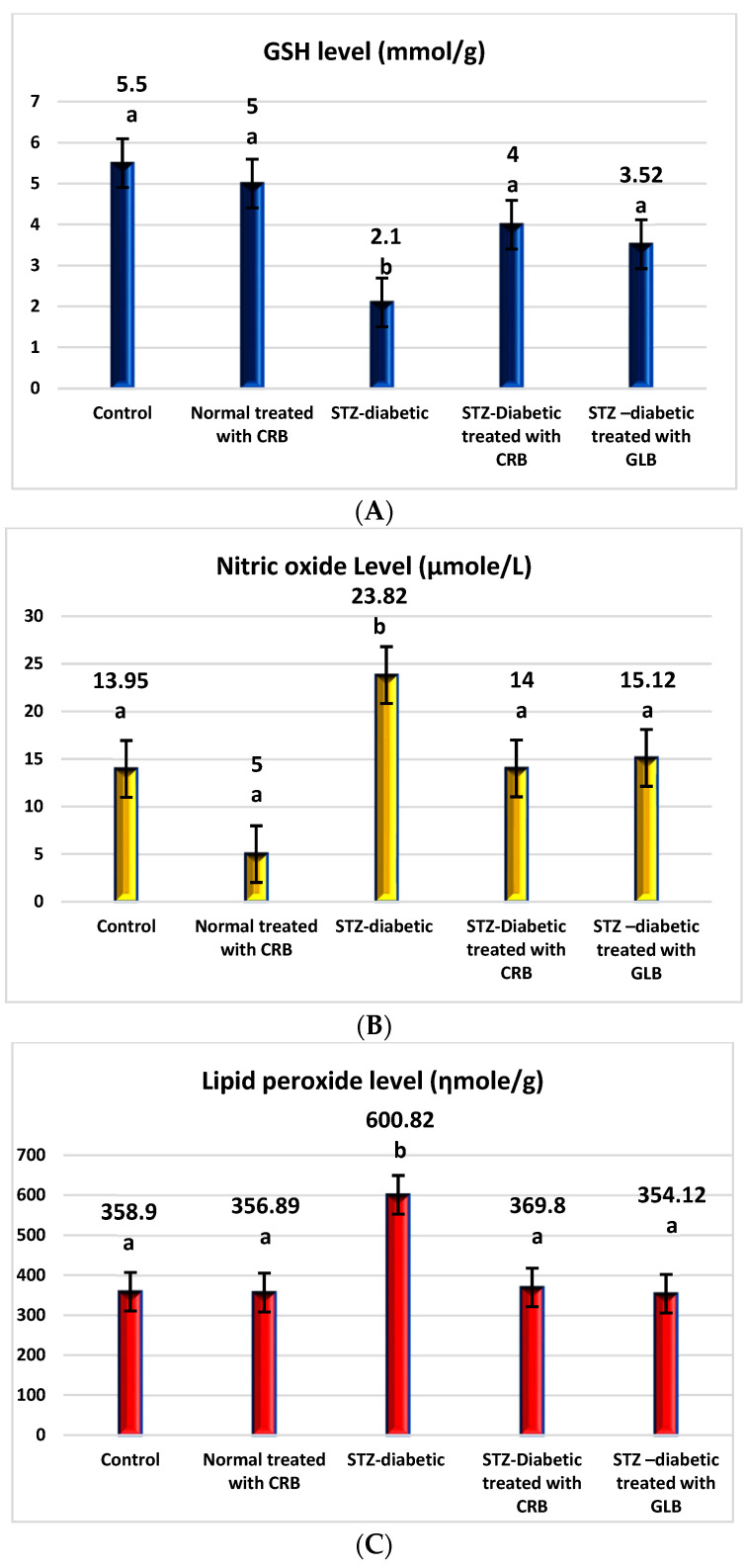

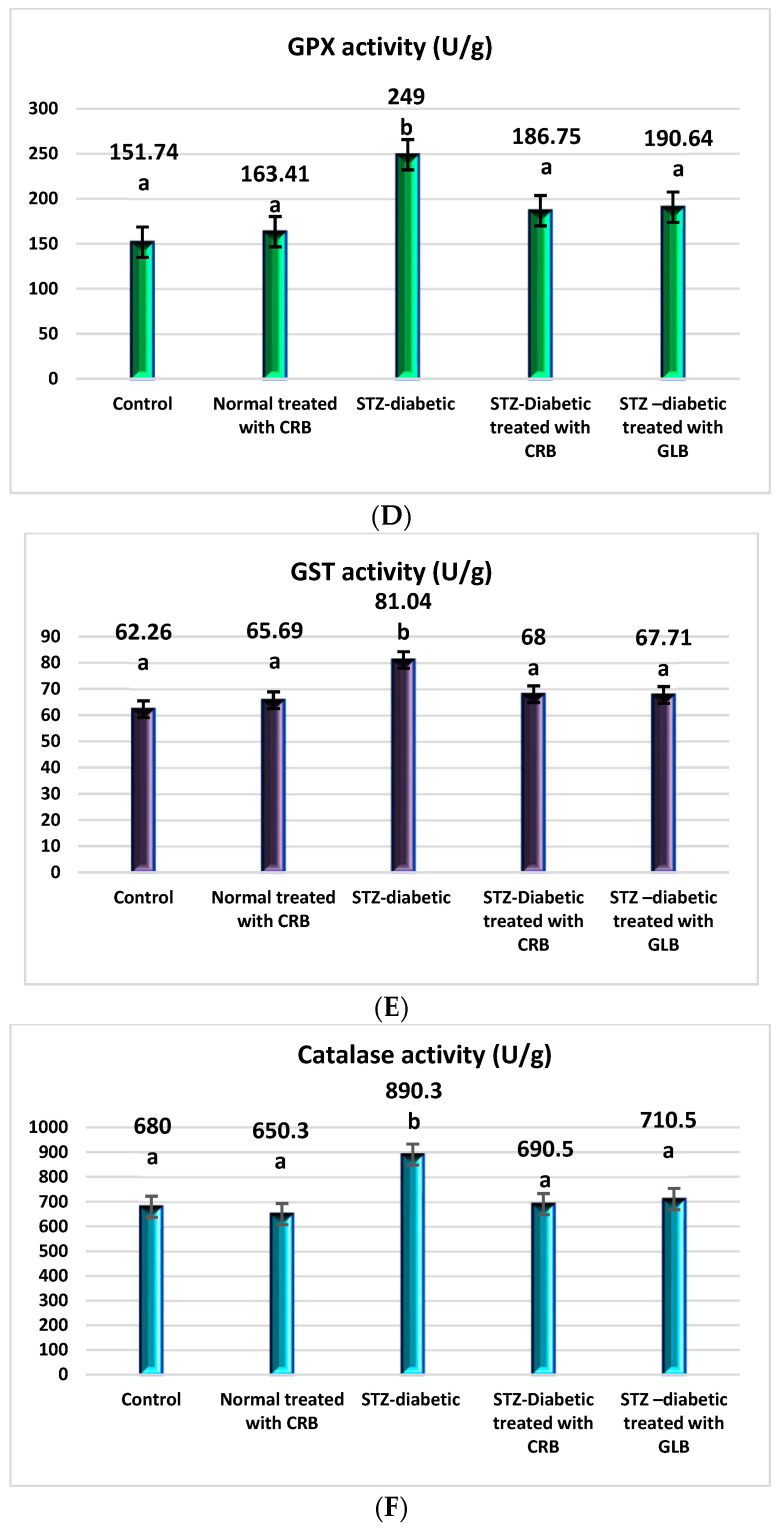
Effect of the n-butanol fraction of *Colvillea racemosa* total extract (CRB) oral administration on oxidative stress markers in Streptozotocin (STZ)-induced diabetic rats; GSH level (**A**), nitric oxide level (**B**), lipid peroxidase level (**C**), GPX activity (**D**), GST activity (**E**), and catalase activity (**F**). Results are displayed as means ± S.D. (measured in triplicate; n = 3). Means with different letters are significantly different (*p* < 0.05).

The raised levels of oxidative stress in diabetic rats are a result of glucose autoxidation, protein glycation, the peroxidation of lipid, and antioxidant enzyme-lowering activities [36]. The diabetic group showed an increase in the levels of nitric oxide, glutathione peroxidase (GPX), glutathione-s-transferase (GST), catalase, and lipid peroxide by 70.75%, 64.09%, 30.16%, 30.93%, and 67.41%, respectively, and a decline in the level of GSH by 61.82% compared with the control group (*p* < 0.05) Figure 3.

In contrast, the oral administration of GLB effectively alleviates oxidative stress, as evidenced by a potent decrease in nitric oxide, GPX, GST, catalase, and lipid peroxide by 36.52%, 23.44%, 16.45%, 20.19%, and 41.06%, respectively, and an increase in GSH level by 67.62% (*p* < 0.05) compared with diabetic rats. Otherwise, the treatment of diabetic rats orally with CRB decreased serum nitric oxide, GPX, GST, catalase, and lipid peroxide levels by 41.23%, 25%, 16.09%, 22.44%, and 38.45%, respectively, and increased the GSH level by 90.48%.

##### In Vivo Inflammatory Markers Assessment Using (STZ) Induced Diabetic Rats

**Figure 4:**
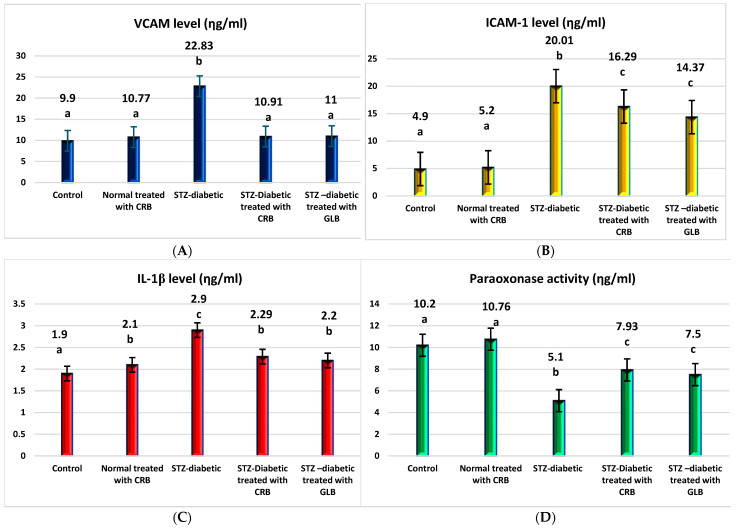

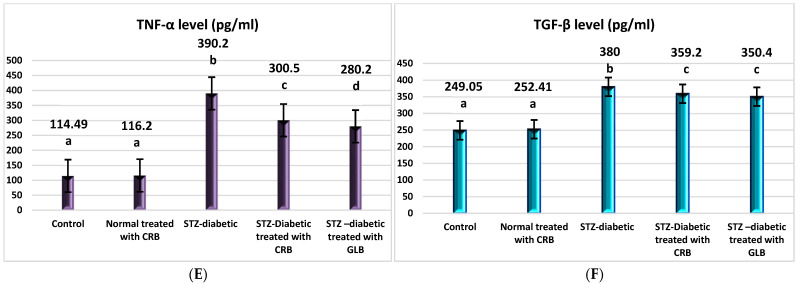
Effect of n-butanol fraction of *Colvillea racemosa* total extract (CRB) oral administration on inflammatory markers in Streptozotocin (STZ)-induced diabetic rats: VCAM level (**A**), ICAM-1 level (**B**), IL1-β level (**C**), paraoxonase activity (**D**), TNF-α level (**E**), and TGF-β level (**F**). Results are displayed as means ± S.D. (measured in triplicate; n = 3). Means with different letters are significantly different (*p* < 0.05).

TNF-α was the first factor at the nexus of inflammation and metabolic illness. Insulin resistance was the cause of increased TNF-α generation in tissue [37]. Compared with the control group, the diabetic showed higher levels of VCAM, ICAM-1, TNF-α, IL1-β, and TGF-β by 130.6%, 308.4%, 240.8%, 52.6%, and 52.6%, respectively, and a decrease in paraoxonase by 50% (*p* < 0.05), as shown in Figure 4.

The oral administration of GLB effectively ameliorated inflammatory markers, manifested as a potent decrease in VCAM, ICAM-1, TNF-α, IL1-β, and TGF-β by 51.8%, 28.2%, 28.2%, 24.1%, and 7.8%, respectively, and an increase in paraoxonase by 47.1% (*p* < 0.05) compared with diabetic rats. However, the treatment of diabetic rats orally with CRB successfully decreased serum VCAM, ICAM-1, TNF-α, IL1-β, and TGF-β by 52.2%, 18.6%, 22.9%, 21.03%, and 5.5%, respectively, and increased paraoxonase by 55.5%.

##### Histopathological Examination

1. Liver

**Figure 5:**
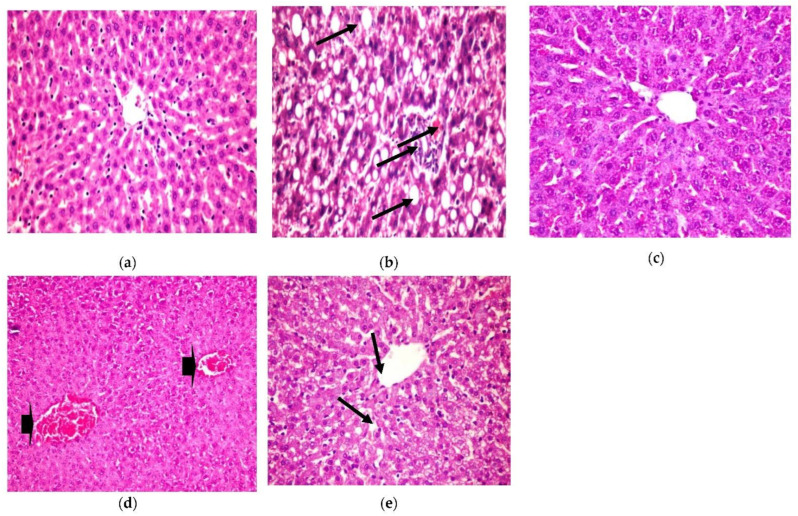
Histopathological examination for (1) liver. (**a**) Liver from the standard group, (**b**) liver from positive diabetic rats, (**c**) liver from the group treated with CRB (Lesion Score: 0), (**d**) liver from the group treated with CRB (Lesion Score: +) and (**e**) liver from the group treated with standard drug (Lesion Score: ++).

The present histopathological examination, as shown in Figure 5, shows liver from a photomicrograph of a control healthy rat (a), while in photomicrograph (b) is shown liver from a positive diabetic rat with diffused fatty degenerated hepatocytes, which appeared as circumscribed vacuolated cells accompanied with the appearance of a signet ring (H&E × 400) (Lesion Score: ++++). However, the photomicrograph of diabetic rats treated with CRB (c) showed the complete disappearance of diabetic lesions, with apparently healthy hepatic parenchyma with normal hepatocytes, blood sinusoids, and portal area (H&E × 400) (Lesion Score: 0). Nevertheless, in other liver sections of diabetic rats treated with CRB, photomicrograph (d) shows the congestion of the central vein (Lesion Score: +). Furthermore, photomicrograph (e) displays the moderate loss of diabetic lesions and small numbers of vacuolated hepatocytes in diabetic rats treated with GLB (Lesion Score: ++). By comparing liver photomicrographs of CRB- and GLB-treated groups, we found a similar effect. Previous research is consistent with the current findings [38,39].

2. Pancreas

**Figure 6:**
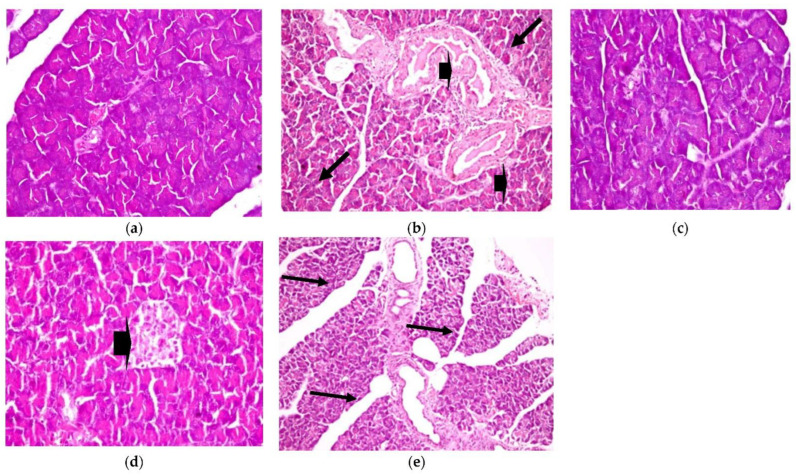
Histopathological examination for **(2)** pancreas. (**a**) Pancreas from the standard group (Lesion Score: 0), (**b**) pancreas from the positive diabetic group (Lesion Score: ++++), (**c**) pancreas from the group treated with CRB (Lesion Score: 0), (**d**) pancreas from the group treated with CRB (Lesion Score: +) and (**e**) pancreas from the group treated with standard drug (Lesion Score: ++).

Similarly, histopathological observation, as shown in Figure 6, shows a healthy control pancreas photomicrograph (a). However, photomicrograph (b) shows a diabetic pancreas with hyperplasia in the pancreatic duct, accompanied by congestion in the blood vessels (H&E X 200) (Lesion Score: ++++). Otherwise, in diabetic rats treated with CRB, as shown in photomicrograph (c), there was complete fading of the diabetic lesions with apparently healthy pancreatic acini, islets, and ducts (H&E X 400) (Lesion Score: 0). Meanwhile, other pancreas sections from diabetic rats treated with CRB, as shown in photomicrograph (d), showed a loss of the diabetic lesions with slight hyperplasia of the pancreatic islets (arrowhead) (H&E X 400) (Lesion Score: +). However, diabetic rats treated with GLB, as shown in photomicrograph (e), showed moderate fading of the diabetic lesions with dilated pancreatic ducts (H&E X 200) (Lesion Score: ++). By comparing pancreas photomicrographs of CRB- and GLB-treated groups, we found that CRB improves the diabetic features of the pancreas. Prior research has found vacuolation of cells in the islets of Langerhans and focal bleeding in the pancreas of diabetic rats, in addition to hyperplasia in the pancreatic duct in STZ-induced diabetic rats, as well as significantly dilated and congested blood vessels (Lesion Score: ++++), which are compatible with the current findings [38,39].

3. Kidney

**Figure 7:**
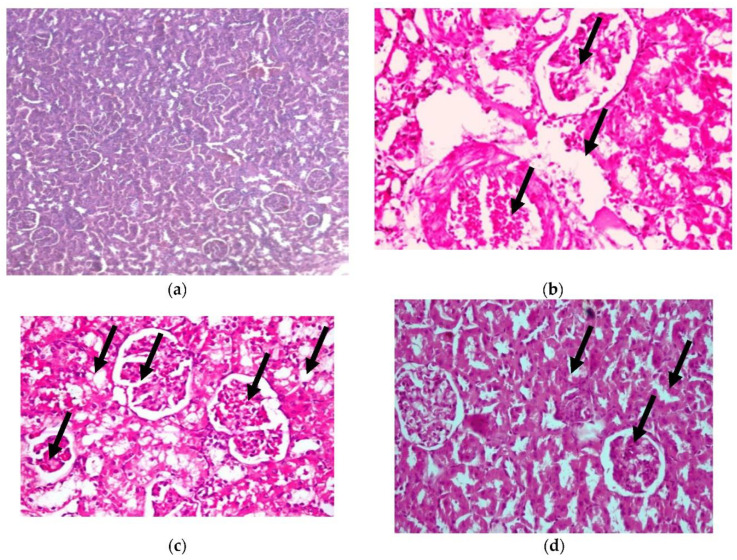
Histopathological examination for (**3**) kidney. (**a**) Photomicrograph of hematoxylin-and-eosin-stained section of normal renal rats, (**b**) photomicrograph of the group of diabetic renal rats treated with CRB (Lesion Score: +), (**c**) photomicrograph of renal diabetes-positive rats (Lesion score +++) and (**d**) photomicrograph of renal diabetic rats treated with standard drug (Lesion score+).

In addition, histopathological examination of the kidney, as shown in Figure 7, shows congestion in the interstitial blood vessel with a thickened wall (arrowhead), together with degenerative changes in the renal tubules (H&E X 400) (as shown in photomicrograph (c) of the renal diabetes-positive group) (Lesion score +++). Additionally, photomicrograph (b) of the kidney cells of diabetic rats in our CRB-treated group shows congestion in the glomerular capillaries (arrows) in addition to the vacuolated renal tubular epithelium (arrowhead) (H&E X 400) (Lesion Score: +). Photomicrograph (d) of renal diabetic rats treated with GLB displays vacuolated glomerular epithelium (arrows) (H&E X 400) (Lesion score+). In parallel results, previous studies are consistent with the current findings [38,39].

Accordingly, the results obtained from the treatment of the diabetic rats with CRB are close to the normal group and the STD-treated diabetic group. It is obvious that CRB effectively ameliorates hyperglycemia and its complications, via inducing pancreatic activity, improving the lipid profile, controlling liver functions, enhancing kidney functions, decreasing oxidative stress, and controlling inflammation, which is the most relied upon for its phytoconstituents. It is the first time for *Colvillea racemosa* leaves to be reported as antihyperglycemic and anti-inflammatory. This could be attributed to its phenolic content [5]. Although *Colvillea racemosa* stems were previously demonstrated to contain naringenin and fisetin flavonoids, nothing was reported about its antihyperglycemic activity [6,40]. However, the *Delonix* species, which belongs to the same subfamily, showed antihyperglycemic, antioxidant, and anti-inflammatory activity [41,42].

### 2.3. Phytoconstituents Investigation Using UPLC-ESI-QTOF-MS of CRB

**Figure 8:**
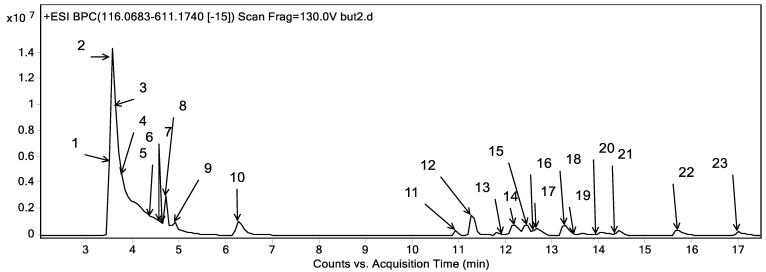
Base peak chromatogram of the n-butanol fraction of *Colvillea racemosa* total extract (CRB) in positive mode at wavelength 250 nm.

Metabolites profiling of the CRB was performed by UPLC-ESI-QTOF-MS and tandem MS/MS. Twenty-three chromatographic peaks were identified, where Figure 8 represents the base peak chromatogram of CRB with the characterized metabolites’ peak numbers.

Metabolite assignments were achieved by the observation of the retention time (RT) for each candidate alongside the mass to charge (*m*/*z*) observed in the positive mode [M + H]^+^, molecular formula, mass score, error, and major MS/MS fragments. The observed values were compared with databases [43,44] and the literature through the Egyptian Knowledge Bank [45], as shown in Table 6. In this context, 23 metabolites were detected. They were classified into phenolic compounds (**13**) and amino acids (**10**).

#### 2.3.1. Phenolic Compounds

The detected phenolic compounds were grouped into flavonoids and phenolic acids. The characterized flavonoids (10) belonged to the flavones subclass and they were of apigenin and luteolin derivatives. As for apigenin derivatives, peak 23 expressed *m/z* 271.06 with a molecular formula of C_15_H_10_O_5_ and was characterized as apigenin. Besides, peaks 15 and 18 revealed the *m/z* 433.11 and molecular formula C_21_H_20_O_10_. They were described as apigenin hexoside. The fragmentation patterns of both isomers were different as the first one showed the neutral loss of two water moieties followed by the consecutive loss of two CHOH moieties (*m/z* 367.08 and 337.07) representing the typical fragmentation pattern of C-glycosides [48] and it was described as apigenin C-hexoside as shown in Figure S1 which was described in the stems of *Colvillea racemosa* as vitexin and isovitexin [6]. Where the seconded isomer exerted the neutral loss of 162 Da complying with the loss of a hexosyl moiety and reflecting O-glycosylation and hence it was described as apigenin O-hexoside which was described before in Fabaceae [43]. It bears noting that both isomers represent 26% of the total characterized phenolics in terms of relative abundance. Moreover, peaks 13 and 17 expressed *m/z* of 579.17 and a molecular formula of C_27_H_30_O_14_ with the sequential loss of a deoxyhexose and a hexose followed by the fragment *m/z* 153 accounting for the ion (^1,3+^A)^+^. They are described as apigenin *O*-deoxyhexoside hexoside (I-II) as shown in Figure S1. 

With regards to luteolin derivatives, peak 22 showed an *m/z* 287.06 and a molecular formula of C_15_H_10_O_6_ with the ion *m/z* 153, 135, and 121 expressing the ions (^1,3+^A)^+^ and its dehydrated form followed by (^1,3+^B)^+^, respectively. It was characterized as luteolin [48]. In this line, peak 16 showed a similar fragmentation pattern to luteolin with an additional loss of a hexosyl moiety and was described as luteolin O-hexoside [43,48]. In addition, peaks 12 and 14 showed an *m/z* 595.17 and molecular formula C_27_H_30_O_15_. The first isomer expressed the typical fragmentation of C-glycosides Table 6 and hence it was described as luteolin C-deoxyhexoside C-hexoside as shown in Figure S1 whereas the former isomer expressed the neutral loss of 146 and 162 Da, respectively representing the O-glycosylation with a deoxyhexoside and a hexose and it was described as luteolin O-deoxyhexoside O-hexoside

They represent the major phenolic compounds with a relative abundance of 31.31% of the total characterized phenolics. In this sense, peak 11 showed an *m*/*z* 611.16 and a molecular formula of C_27_H_30_O_16_, exerted a neutral loss of a water moiety (*m*/*z* 593.15) as well as CHOH moieties (*m*/*z* 575.14, 515.12, 473.11 and 353.06), indicating the presence of C-glycosides [44,48], and was characterized as luteolin di C-hexoside.

Concerning phenolic acids, three derivatives were observed in the characterized metabolites of the CRB of *Colvillea racemosa* leaves extract. Briefly, peak 19 expressed an *m/z* of 285.0947 and a molecular formula of C_13_H_16_O_7_. It exerted the neutral loss of a hexosyl moiety (162 Da) with the appearance of the molecular ion of benzoic acid (*m*/*z* 123), followed by its decarboxylation (*m*/*z* 79). Consequently, it was characterized as benzoyl hexoside, which was found for the first time in the Fabaceae family in accordance with the Reaxys database [44].

Moreover, peak 20, with *m*/*z* 373.15 and molecular formula C_17_H_24_O_9_, expressed decarboxylation followed by the neutral loss of two methyl moieties, accompanied by the loss of a hexosyl moiety, as shown in Figure S2. It was characterized as a syringin. Additionally, peak 21 showed a loss of hexosyl moiety followed by the loss of ethyl and water moieties, as shown in Table 6. It was characterized as ethyl ferulate hexoside, which was reported for the first time in the family Fabaceae according to the Reaxys database [44].

#### 2.3.2. Amino Acids

Regarding amino acids, ten amino acids were observed, as shown in Table 6. Peaks 3, 4, and 5 displayed *m*/*z* 118.09 and a molecular formula of C_5_H_11_NO_2_ and exerted the neutral loss of water, CO_2_ and methyl. They were characterized as hydroxymethyl hydroxypyrrolidine I-III. They were found before in Fabaceae according to the KNApSAcK-Core-System database [43]. In this sense, Figure S2 describes the fragmentation pattern of hydroxymethyl hydroxypyrrolidine III. Peaks 2 and 6 expressed *m*/*z* 146.08 and molecular formula C_6_H_11_NO_3_, and they were characterized as hydroxypipecolic acid I-II. Peak 1 was characterized as proline (*m*/*z* 116.06, C_5_H_9_NO_2_) with a fragment of *m*/*z* 99.05 accounting for deamination [47]. In addition, peaks 8 and 9 were characterized as leucine/isoleucine I-II with a common fragment of *m/z* 86 accounting for the neutral loss of CO_2_ + 2H. In addition, peak 7 expressed a similar fragmentation pattern of leucine/isoleucine with a former loss of a hexosyl moiety, and hence was described as leucine/isoleucine hexoside. Peak 10 with *m*/*z* 166.09 and molecular formula C_9_H_11_NO_2_ applied a neutral loss of NH_3_ and CO_2_, expressing the typical fragmentation of amino acids [47]. Therefore, it was characterized as phenylalanine, as shown in Figure S2.

Finally, the metabolic profiling of CRB may explain the antidiabetic and anti-inflammatory activity of CRB, in which flavonoids are antioxidant molecules found in nature that have anti-diabetic properties. Likewise, growing scientific evidence suggests that they have anti-inflammatory and anti-oxidant properties [51]. The anti-diabetic activity of apigenin and luteolin derivatives was previously assessed through a reduction in gluconeogenic and lipogenic capacity, even with the inhibition of the PKB/AKT pathway [52]. Although apigenin and its derivatives reduce cellular free radicals, prevent cell destruction in pancreatic β-cells, and promote GLUT4 translocation, lowering the glucose level, luteolin and its derivatives increase insulin secretion by suppressing Maf A through the NF-κB signaling cycle and activate PPAR-γ, which targets adiponectin, leptin and GLUT4 genes [53]. In addition, the antidiabetic effect of proline and phenylalanine was revealed by improved glucose uptake through the AMPK pathway [54]. Leucine administration improves glycemic control in humans and rodents with type II diabetes and decreases oxidative stress. CRB’s hypoglycemic, anti-inflammatory, and antioxidant therapeutic properties could be linked to the metabolites discovered in this study.

## 3. Materials and Methods

### 3.1. Plant Material

#### 3.1.1. Collection of Plant

The leaves of *Colvillea racemosa* L., family Fabaceae, were collected from ZOO 30°01′30.7″ N 31°12′57.7″ E Giza, Egypt, during June 2018. Plant authentication was carried out by Prof. Dr. Reem Hamdy (Professor of Plants and Flora, Plants Department, Faculty of Sciences, Cairo University). Voucher specimen no. SAA-180 is saved at the Herbarium of the Department of Pharmacognosy, Faculty of Pharmacy, Suez Canal University. The leaves were air-dried in a dark dry room with humidity control at room temperature, then powdered using a herbal grinding machine.

#### 3.1.2. Preparation of *Colvillea racemosa* Leaf Extract

The dried powdered leaves (1100 g) were macerated in 70% ethanol (to enhance the extraction of many high-polar or medium-to-low-polar compounds by increasing cell wall permeability), then divided into 22 muslin bags, each bag containing 50 g. Two bags each were put in a jar containing 500 mL of 70% ethanol for 5 days under ultrasonic irradiation 1 h per day, for a period of extraction of 20 days till exhaustion. Extracts were collected, filtered, pooled, then evaporated under vacuum (at 50 °C) to give a dark brown residue (110 g). A total of 80 g of extract was fractionated using different solvents of increasing polarity (petroleum ether, methylene chloride, ethyl acetate, *n*-butanol saturated with water, and methanol) to ensure extraction of many compounds.

The extracting solvents were evaporated under vacuum at a temperature not exceeding 50 °C to yield the corresponding *Colvillea racemosa* leaf extractives, CRP (6.23 g), CRMC (12.34 g), CREA (2.54 g), CRB (45.32g), and CRME (11.32g). All extractives were kept for further examination

### 3.2. Proximate Composition and Mineral Content

Proximate analysis of the powdered leaves was performed by adopting the procedures of the A.O.A.C. [55], which include: determination of moisture content, total ash content, total lipid, and crude fiber. Total protein was detected by the Kjeldahl method [56]. Meanwhile, the content of total carbohydrate for the leaves was determined according to Kostas [57]. The nutritive value was stated in kilocalories per 100 g of the dry weight of air-dried leaves, measured by Formula (1) [58].

Nutritive value = (4 × %protein) + (9 × %crude fat) + (3.75 × %total carbohydrate).(1)

The macro- and micro-minerals were estimated by inductively coupled plasma atomic emission spectroscopy (ICP-AES), Thermo Sci, model: iCAP6000 series [59]. Na and K were determined by atomic absorption spectrometry [60]. The powdered sample was introduced into pressure vessels and digested under a controlled temperature and pressure. The concentrations of Ca, Mg, Cd, Cu, Pb, Mn, Se, and Zn were detected. Argon gas was used for the excitation of the element atoms.

### 3.3. Kits and Chemicals for Biological Assessment

GLB (glibenclamide), STZ, α-glucosidase (*Saccharomyces cerevisiae*), and 3,5,di-nitrosalicylic acid (DNS) were acquired from Sigma Aldrich (St. Louis, MO, USA). Pancreatic function kits, lipid profile kits, liver function kits, kidney function kits, antioxidants, and oxidative stress biomarkers were obtained from Bio-diagnostic (Egypt). Adhesion molecules (VCAM-1 and ICAM-1), and inflammatory biomarker (IL-1β, TNF-α, and TGF-β1) assay kits were obtained from Cusabio, Wuhan, China. Paraoxonase assay was obtained from BioSource Canada. *P*-nitro-phenyl-α-D-glucopyranoside (*p*-NPG), sodium carbonate (Na_2_CO_3_), sodium dihydrogen phosphate, and disodium hydrogen phosphate were acquired from Hi-Media. The remaining chemicals were of the best quality and are available on the market.

### 3.4. Antihyperglycemic and Anti-Inflammatory Activity

#### 3.4.1. In Vitro Anti-Hyperglycemic Assessment via Inhibition of α-Amylase Activity Assay

The 3,5-dinitrosalicylic acid (DNSA) technique was used to perform the α-amylase inhibition assay [61]. A total of 1mL of the extract at various concentrations (1000−7.81 μg/mL) was added to 1ml of enzyme solution (which was prepared by dissolving enzyme in a 20 mM phosphate buffer (pH = 6.9) at the concentration of 0.5 mg/mL), then mixed and incubated at 25 °C for 10 min. This was followed by the addition of 0.5% starch solution (1 mL) to the mixture, then incubated for 10 min at 25 °C. Finally, 2 mL of dinitro salicylic acid (DNS, color reagent) was added to terminate the reaction, then the mixture was heated in a boiling water bath (5 min). The absorbance was detected colorimetrically at 565 nm after cooling. The inhibition % was calculated by Formula (2).

%inhibition = (1 − As/Ac) × 100(2)

Ac is the absorbance of the control reaction and As is the absorbance of the test sample. Acarbose was the standard drug.

#### 3.4.2. In Vitro Anti-Hyperglycemic Assessment Using Inhibition of α-Glucosidase Activity Assay

α-Glucosidase inhibitory activity was detected according to the reference technique with slight adjustment [62]. In total, 10 μL of the α-glucosidase enzyme at a concentration of 1 U/mL was added to 50 μL phosphate buffer (100 mM, pH = 6.8), and 20 μL of different concentrations of samples (1000 to 7.81 μg/mL) were preincubated at 37 °C for 15 min. At the same time, 20 μL P-NPG (5 mM) was added as a substrate and incubated at 37 °C for 20 min. Finally, the reaction was terminated by 50 μL Na_2_ CO_3_ (0.1 M). The absorbance of the released *p*-nitrophenol was detected at 405 nm using a Multiplate Reader. Acarbose was encompassed as a standard. The percentage inhibition was calculated using Formula (2).

The IC_50_ value was defined as the concentration of α-amylase/α-glucosidase inhibitor required to inhibit 50% of its activity under the assay conditions.

#### 3.4.3. In Vitro Anti-Inflammatory Evaluation Using Membrane Stabilization % Assay

Erythrocyte suspension preparation: total blood was taken from rats through cardiac puncture with heparinized syringes. Three washes with 154 mM NaCl isotonic buffered solution in 10 mM sodium phosphate buffer (pH 7.4) were performed on blood. Finally, blood was centrifuges for 10 min at 3000× *g*.

Hypotonic-solution-induced erythrocyte hemolysis was used to assess the samples’ membrane-stabilizing activity. The test sample was composed of 0.5 mL of stock erythrocyte suspension combined with 5 mL of hypotonic solution (50 mM NaCl) in 10 mM sodium phosphate-buffered saline (pH 7.4) containing the extract (1000–7.81 g/mL) or indomethacin. The control consisted of 0.5 mL of RBC mixed with hypotonic-buffered saline solution on its own. The mixtures were incubated at room temperature for 10 min before being centrifuged at 3000× *g* for 10 min. The absorbance of the supernatant was measured at 540 nm in 96-well plates. According to the modified approach presented, the % suppression of hemolysis or membrane stabilization was calculated using Formula (3) [63].

Inhibition of hemolysis (membrane stabilization %) = 100 × (OD1 − OD2/OD1%(3)

where OD1 is the optical density of hypotonic-buffered saline solution alone, and OD2 is the optical density of the test sample in a hypotonic solution.

The IC_50_ value was defined as the concentration of the sample to inhibit 50% RBCs hemolysis under the assay conditions.

#### 3.4.4. In Vivo Anti-Hyperglycemic Activity Evaluation in Rats

##### Rats and Rat Treatment

For this study, fifty male albino rats weighing from 200 ± 50 g were divided into 5 groups of 10 rats each. Normal control rats, group 1, were merely given a citrate buffer. Normal healthy rats, group 2, were orally administrated CRB (0.5 g/kg bodyweight every day for 30 days; according to LD_50_ study).

STZ diabetic rats comprised group 3, injected intraperitoneally with one dose of 60 mg/kg of STZ. Group 4 comprised STZ diabetic rats given CRB orally at a dose 0.5 g/kg body weight for 30 days; each rat received 55 mg/0.5 mL distilled water [64]. Group 5 was the positive control, where diabetic rats were administered 10 mg/kg body weight orally (each rat received 1.5 mg/0.5 mL distilled water) of GLB. The study was carried out in accordance with the Canadian Council on Animal Care’s guidelines. The ethics committee at the Faculty of Pharmacy, Suez Canal University, Egypt approved the experimental protocol (Code # 201903PHDA1).

##### Induction of Hyperglycemia in Rats

STZ was used to produce type II diabetes in rats, which was given intraperitoneally with a single dose of STZ (60mg/kg body weight), dissolved before use in 0.01 M citrate buffer [65]. Rats had unrestricted access to food and water following injection. After two hours each rat was given 2.5 mL of 40% glucose solution in addition to 5% glucose solution orally to consume overnight to counter hypoglycemic shock [66]. For the experiment, hyperglycemic rats had blood glucose levels ≥ 250. GLB, standard oral hypoglycemic, was used as a positive control [67].

##### Preparation of Sample for Analysis

- Serum

First, the weight of each rat was measured, then a blood sample taken in a dry, clean test tube via puncturing the sublingual vein, which was allowed to clot for 10 min, then centrifuged at 3000 rpm to separate the serum, which was kept at −80 °C until analysis.

- Tissue

First, the weight of the liver tissue was measured, then the tissue was homogenized using a polytron homogenizer with 10 volumes of a suitable medium. Then, the supernatant was kept at −80 °C for assessment. The extraction technique used 1 g of tissue that was homogenized in 9 mL physiological saline (0.9N). The tissue sample was centrifuged for 10 min at 3000 rpm and a collection of supernatant was used for various biochemical tissue analysis [68].

##### Detection of the Biochemical Parameters

1. Assessment of Fasting Blood Glucose (FBG) and α-amylase activity

Serum glucose was measured [69]; meanwhile, α-amylase was determined in serum [70].

2. Assessment of Lipid Profile

Serum total lipid and triglyceride were determined colorimetrically [71,72], while total cholesterol and HDL-cholesterol were investigated by enzymatic procedures [73,74].

3. Assessment of liver function

Serum AST and ALT were detected colorimetrically [75]. ALP activity was determined according to the modified method [76]. Total bilirubin and total protein were determined according to spectrophotometric procedures [77,78].

4. Assessment of kidney function

Serum content of urea and creatinine were estimated [79,80].

5. Assessment of antioxidants and oxidative stress biomarkers

Reduced glutathione concentrations in the samples were determined spectrophotometrically [81]. Nitric oxide was measured using the colorimetric method in tissue liver homogenate [82]. GPX was assayed [83]. GST activity was determined with 1-chloro-2,4-dinitrobenzene (CDNB) at 340 nm [84]. Catalase activity was determined by the colorimetric method with hydrogen peroxide as substrate [85]. Serum lipid peroxides were calorimetrically measured using the thiobarbituric acid reaction method [86].

6. Immunosorbent assay

ELISA (a sandwich enzyme immunoassay) was used to measure adhesion molecules (ICAM-1 and VCAM-1), inflammatory biomarkers (TGF-β1, IL-1β, and TNF-α) level, and the level of anti-inflammatory enzyme (paraoxonase) in serum [87].

7. Histopathological examination

The sacrificed rats’ organs (pancreas, liver, and kidneys) were removed and immersed in a solution of 10% formalin. Then, the specimens were cut, rinsed, and dehydrated in a progressively higher quality of alcohol. For histopathological analysis, dehydrated specimens were cleansed in xylol, embedded in paraffin, sectioned at 4-6 µm thickness, and stained with hematoxylin and eosin [88].

### 3.5. UPLC-ESI-QTOF-MS Analysis

LC/QTOF (6530, Agilent Technologies, Santa Clara, CA, USA) was accompanied with an autosampler—G7129A, a quat. pump (G7104C), and a column comp (G7116A) at Fayoum University, Faculty of Pharmacy. The LC-ESI-QTOF-MS investigation was performed on a Poroshell 120 EC-C18, 1000 bar, RP-18 column from Agilent Technologies, dimensions: 150 mm × 3 mm, dp = 2.7 µm. In a flow rate of 0.2 mL /min, a combination of solvent A and solvent B was used as the mobile phase, A: water + 0.1 percent formic acid, and B: acetonitrile + 0.1 percent formic acid, in which isocratic elution from 0 to 2 min, 10% B, linear elution (2–5 min) from 10 to 30% B, and gradient elution (5–15 min) from 30 to 70% B, were followed with gradient elution (15–18 min) from 70 to 100% B and finally, gradient elution (18–20 min) from 100 to 10% B. The sample was ready at a concentration of 1 mg/mL in HPLC-grade methanol, and the injection volume was 10 µL. The metabolites characterization was according to the strategy proposed by several studies [47,49,89]. Metabolite assignments were achieved by the observation of the retention time (RT) for each candidate alongside the mass to charge (*m*/*z*) observed in the positive mode [M+H]+, molecular formula, mass score not less than 80%, error ± 3, and major MS/MS fragments.

- Statistical analysis

The results were statistically analyzed using one-way analysis of variance (ANOVA), in which our findings were displayed as mean ± standard deviation of the mean (SD). Values with *p* < 0.05 were considered to be significantly different [90].

## 4. Conclusions

According to the results of this study, *Colvillea racemosa* leaves have high nutritional value. In addition, CRB showed promising anti-hyperglycemic effects, as shown in in vitro and in vivo investigations. This could contribute to the CRB content of polyphenolic compounds. This could be appreciated by a group of diabetic patients due to the naturally occurring leaves. Nevertheless, extra in vivo investigations and clinical trials for the plant and identified compounds are strongly suggested to authenticate its therapeutic activity as an anti-hyperglycemic and anti-inflammatory agent. Additionally, various pharmaceutical dosage forms containing *Colvillea racemosa* leaves, either in the form of crude extracts or herbal teas, could be formulated after validating their beneficial potential.

## Figures and Tables

**Table 1:** Proximate composition (on a dry matter basis) of *Colvillea racemosa* leaves.

Proximate Composition	(g/100 g)	Proximate Composition	(g/100 g)
Moisture %	8.2 ± 0.4	Total carbohydrate %	49.03 ± 2.1
Ash %	13 ± 1.3	Total lipid %	11.74 ± 0.32
Fiber %	14.44 ± 0.83	Total protein %	26.23 ± 0.55
Energy value (Kcal/100 g)		394.4425 (Kcal/100 g)	

Values are (mean of triplicate ± standard deviation).

**Table 2:** Mineral contents (on a dry matter basis) of *Colvillea racemosa* leaves.

Element	mg/100 g	RDI * (mg)	MDI * (mg)
Na	186.03	1500–2300	2300
Mg	344.01	310–420	350
K	2001.90	4700–5000	N.D
Ca	2426.67	1000–1300	2500
Mn	8.86	1.8–2.3	11
Cu	0.705	0.9	10
Zn	2.965	8–11	N.D
Se	N.D	0.055	0.4
Cd	N.D	0.02	0.02
Pb	N.D	0.0125	0.0125

* RDI: recommended daily intake for adults [17,18]. * MDI: maximum daily intake for adult [17,18]. N.D: not detected.

**Table 3:** Effect of the n-butanol fraction of *Colvillea racemosa* total extract (CRB) orally administered on lipid profile (mg/dL) in Streptozotocin (STZ)-induced diabetic rats.

	Groups	Total Lipid	Triglycerides	Total Cholesterol	HDL-Cholesterol
1	Normal rats	574.11 ± 29.35 ^a^	111.20 ± 2.19 ^c^	140.90 ± 20.05 ^a^	50.10 ± 1.28 ^a^
2	Normal rats treated with CRB	600.11 ± 20.33 ^a^	110.22 ± 9.50 ^bc^	148.20 ± 9.00 ^a^	51.11 ± 1.08 ^a^
3	STZ-treated diabetic rats	910.20 ± 20.00 ^b^	170.92 ± 6.05 ^b^	220.40 ± 8.10 ^b^	30.10 ± 2.00 ^b^
4	STZ—diabetic rats treated with CRB	611.11 ± 39.28 ^a^	135.20 ± 7.13 ^a^	154.90 ± 10.40 ^a^	52.00 ± 2.70 ^a^
5	STZ—diabetic rats treated with GLB	670.70 ± 29.00 ^a^	128.00 ± 8.20 ^ac^	143.00 ± 6.10 ^a^	53.10 ± 2.30 ^a^

Results are displayed as means ± S.D. (measured in triplicate; n = 3). Means with different letters are significantly different (*p* < 0.05).

**Table 4:** Effect of n-butanol fraction of *Colvillea racemosa* total extract (CRB) orally administered on liver function in Streptozotocin (STZ)-induced diabetic rats.

	Groups	ALT	AST	ALP	Bilirubin	Total Protein
1	Normal rats	80.71 ± 2.40 ^a^	144.20 ± 10.55 ^a^	94.71 ± 1.91 ^a^	0.60 ± 0.09 ^a^	5.59 ± 0.21 ^a^
2	Normal rats treated with CRB	83.00 ± 2.60 ^a^	140.50 ± 4.10 ^a^	97.11 ± 1.68 ^a^	0.60 ± 0.07 ^a^	5.24 ± 0.38 ^a^
3	STZ-treated diabetic rats	170 ± 11.00 ^b^	230.54 ± 10.20 ^b^	163.44 ± 15.44 ^b^	1.20 ± 0.10 ^b^	3.67 ± 0.36 ^b^
4	STZ—diabetic rats treated with CRB	96.20 ± 8.30 ^ac^	158.80 ± 5.90 ^a^	99.48 ± 6.60 ^a^	0.70 ± 0.02 ^a^	5.25 ± 0.33 ^a^
5	STZ—diabetic rats treated with GLB	89.22 ± 6.70 ^a^	140.00 ± 8.80 ^a^	96.03 ± 11.50 ^a^	0.65 ± 0.02 ^a^	5.16 ± 0.69 ^a^

Results are displayed as means ± S.D. (measured in triplicate; n = 3). Means with different letters are significantly different (*p* < 0.05).

**Table 5:** Effect of *n*-butanol fraction of *Colvillea racemosa* total extract (CRB) orally administered on kidney function (mg/dL) in Streptozotocin (*STZ*)-induced diabetic rats.

	Groups	Creatinine Level	Urea Activity
1	Normal rats	0.26 ± 0.02 ^b^	32.70 ± 2.52 ^b^
2	Normal rats treated with CRB	0.25 ± 0.09 ^b^	30.95 ± 1.70 ^b^
3	STZ-treated diabetic rats	0.49 ± 0.07 ^a^	60.59 ± 2.06 ^a^
4	STZ—diabetic rats treated with CRB	0.29 ± 0.05 ^b^	36.22 ± 3.32 ^b^
5	STZ—diabetic rats treated with GLB	0.25 ± 0.06 ^b^	33.55 ± 2.72 ^b^

Results are displayed as means ± S.D. (measured in triplicate; n = 3). Means with different letters are significantly different (*p* < 0.05).

**Table 6:** Metabolite profiling of CRB via UPLC-ESI-QTOF-MS in positive mode.

No.	RT (min)	[M + H]^+^	M	Molecular Formula	Score	Error (mDa)	MS/MS	Proposed Compound	Area	Reference
1	3.499	116.0706	115.0633	C_5_H_9_NO_2_	99.21	−0.01	99.0504, 98.0604	Proline	2.14 × 10^6^	[43]
2	3.566	146.0814	145.0741	C_6_H_11_NO_3_	97.69	−0.22	128.0704, 100.0758	Hydroxypipecolic acid I	1.52 × 10^8^	[43]
3	3.634	118.0865	117.0789	C_5_H_11_NO_2_	87.69	−0.27	59.0735	Hydroxymethyl hydroxypyrrolidine I	4.71 × 10^6^	[43]
4	3.768	118.0864	117.0789	C_5_H_11_NO_2_	99.71	−0.16	72.0813, 59.0736	Hydroxymethyl hydroxypyrrolidine II	1.08 × 10^7^	[43]
5	4.374	118.0862	117.0789	C_5_H_11_NO_2_	97.04	0.07	59.0731	Hydroxymethyl hydroxypyrrolidine III	1.81 × 10^6^	[43]
6	4.576	146.0816	145.0741	C_6_H_11_NO_3_	97.23	−0.46	128.0702, 100.0756	Hydroxypipecolic acid II	4.80 × 10^7^	[43]
7	4.644	294.155	293.1477	C_12_H_23_NO_7_	99.29	0.12	132.1016, 86.0966	Leucine/Isoleucine hexoside	5.13 × 10^6^	[46]
8	4.711	132.1019	131.0945	C_6_H_13_NO_2_	99.94	−0.02	86.0966	Leucine/Isoleucine I	1.34 × 10^7^	[47]
9	4.913	132.1021	131.0945	C_6_H_13_NO_2_	99.5	−0.2	86.0967	Leucine/Isoleucine II	1.03 × 10^7^	[47]
10	6.26	166.0867	165.0793	C_9_H_11_NO_2_	98.66	−0.36	149.0597, 121.0836, 120.0807, 105.0710	Phenylalanine	1.43 × 10^7^	[43]
11	10.905	611.1618	610.1543	C_27_H_30_O_16_	97.7	−0.92	593.1504, 575.1391, 515.1195, 473.1074, 353.0630	Luteolin di *C*-hexoside	2.19 × 10^6^	[43,48]
12	11.275	595.1679	594.1585	C_27_H_30_O_15_	93.44	−1.93	577.1558, 559.1454, 475.1237, 457.1130, 335.0805, 307.0607, 137.1056, 135.0897, 133.0836	Luteolin *C*-deoxyhexoside *C*-hexoside	1.29 × 10^7^	[48]
13	11.951	579.1725	578.1653	C_27_H_30_O_14_	90.32	−1.72	N.D.	Apigenin *O*-deoxyhexoside hexoside I	8.18 × 10^5^	[43]
14	12.184	595.1682	594.1585	C_27_H_30_ O_15_	91.47	−2.19	449.1085, 287.0549, 153.0187, 137.0227	Luteolin *O*-deoxyhexoside -hexoside	9.25 × 10^6^	[48]
15	12.454	433.1142	432.1062	C_21_H_20_O_10_	96.03	−1.23	415.1026, 397.0927, 367.0809, 337.0705, 119.0506	Apigenin *C*-hexoside	9.40 × 10^6^	[49]
16	12.588	449.1087	448.1015	C_21_H_20_O_11_	97.24	−0.98	287.054,153.0189,135.0438,121.0623	Luteolin *O*-hexoside	3.31 × 10^6^	[48]
17	12.652	579.1725	578.1653	C_27_H_30_O_10_	95.91	−1.38	431.1131, 271.0599 153.0174	Apigenin *O*-deoxyhexoside hexoside II	4.90 × 10^6^	[43]
18	13.262	433.1142	432.1062	C_21_H_20_O_10_	98.94	−1.25	271.0600, 153.0181, 119.0484	Apigenin *O*-hexoside	9.21 × 10^6^	[48]
19	13.463	285.0947	284.0874	C_13_H_16_O_7_	84.36	1.91	123.0183, 79.0543	Benzoyl hexoside	2.70 × 10^6^	[50]
20	13.935	373.1472	372.1403	C_17_H_24_O_9_	90.71	1.69	329.0868, 299.1014, 211.0896	Syringin	3.36 × 10^6^	[43]
21	14.339	385.1463	384.1399	C_18_H_24_O_9_	86.72	2.15	223.1290, 221.0660, 173.0804	Ethylferulate hexoside	3.53 × 10^6^	[44]
22	15.753	287.0552	286.048	C_15_H_10_O_6_	99.71	−0.24	153.0189, 135.0438, 121.0623	Luteolin	5.70 × 10^6^	[48]
23	16.695	271.0602	270.0528	C_15_H_10_O_5_	99.24	−0.18	N.D.	Apigenin	3.37 × 10^6^	[48]

N.D., non-detected.

## Data Availability

This work contains all of the data.

## Supplementary Materials

The following supporting information can be downloaded at: https://www.mdpi.com/article/10.3390/plants11060830/s1, Figure S1: Fragmentation patterns of (a) apigenin C hexoside, (b) apigenin O-deoxyhexoside hexoside II, and (c) luteoin C-deoxyhexoside C-hexoside; Figure S2: Fragmentation pattern of (a) syringin, (b) hydroxymethyl hydroxypyrrolidine III, and (c) phenylalanine.

## Abbreviations

CRE	*Colvillea racemosa* ethanol extract
CRP	petroleum ether fraction of *Colvillea racemosa* ethanol extract
CRMC	methylene chloride fraction of *Colvillea racemosa* ethanol extract
CREA	ethyl acetate fraction of *Colvillea racemosa* ethanol extract
CRB	*n*-butanol fraction of *Colvillea racemosa* ethanol extract
CRME	methanol fraction of *Colvillea racemosa* ethanol extract
STZ	streptozotocin
GSH	glutathione
UPLC-ESI-QTOF-MS	ultra-performance liquid chromatography–electrospray ionization quadrupole time-of-flight mass spectrometry
Kcal	kilocalories
Ros	reactive oxygen species
GLB	glibenclamide
A.O.A.C	Association of Analytical Chemists
