# Supplementary material for: Quality Control, Anti-Hyperglycemic, and Anti-Inflammatory Assessment of Colvillea racemosa Leaves Using In Vitro, In Vivo Investigations and Its Correlation with the Phytoconstituents Identified via LC-QTOF-MS and MS/MS
Source: Plants (Basel). 2022 Mar 21;11(6):830. doi: 10.3390/plants11060830 (PMC8948708; doi:10.3390/plants11060830)
Supplement: Supplementary file 1 [file plants-11-00830-s001.zip › plants-1641468-supplementary/plants-1641468 supplementary figure caption.pdf]

Figure S1: Fragmentation patterns of (**a**) apigenin C hexoside, (**b**) apigenin O-deoxyhexoside hexoside II, and (**c**) luteoin C-deoxyhexoside C-hexoside.
Figure S2: Fragmentation pattern of (**a**) syringin, (**b**) hydroxymethyl hydroxypyrrolidine III, and (**c**) phenylalanine.
